# Comparative Evaluation of the Vasodilatory Effects of Desflurane, Isoflurane, and Sevoflurane Using Perfusion Index During General Anaesthesia

**DOI:** 10.7759/cureus.116736

**Published:** 2026-09-22

**Authors:** Anshita Dabur, Nigar Bari, Kanika Sachdeva, Nishit Govil

**Affiliations:** 1 Department of Anesthesiology, Shri Guru Ram Rai Institute of Medical and Health Sciences, Dehradun, IND

**Keywords:** desflurane, isoflurane, perfusion index, sevoflurane, vasodilation

## Abstract

Background

Volatile inhalational anaesthetic agents produce changes in peripheral vascular tone and systemic haemodynamics. Perfusion index (PI), derived from the pulse oximetry signal, provides a non-invasive measure of peripheral perfusion and can be used to assess changes in peripheral vascular tone. This study was undertaken to compare the vasodilatory effects of desflurane, isoflurane, and sevoflurane using PI as the primary outcome measure and to evaluate associated haemodynamic changes.

Methods

This prospective comparative study included 120 patients (40 each in desflurane, isoflurane, and sevoflurane groups) aged 18-60 years, American Society of Anesthesiologists (ASA) I-II, undergoing elective surgery under general anaesthesia. Anaesthesia was induced with fentanyl 2 µg/kg and propofol 2 mg/kg, with vecuronium 1 mg/kg for airway management, and maintained with the assigned volatile agent at 1.0 minimum alveolar concentration (MAC) without nitrous oxide or other anaesthetic adjuvants. PI was recorded at baseline and at 1, 5, 15, 20, and 30 minutes after induction. Heart rate (HR), systolic blood pressure (SBP), diastolic blood pressure (DBP), and mean arterial pressure (MAP) were also assessed at the corresponding time points. The primary outcome was the difference in PI among the three anaesthetic groups.

Results

Baseline PI was comparable among the desflurane, isoflurane, and sevoflurane groups (1.055 ± 0.136, 1.118 ± 0.153, and 1.112 ± 0.179, respectively; F = 1.960, p = 0.145). Following induction, PI increased significantly in all three groups, with significant between-group differences at every assessed post-induction time point. At 1 minute, PI was 2.753 ± 0.377, 2.493 ± 0.241, and 2.445 ± 0.264 in the desflurane, isoflurane, and sevoflurane groups, respectively (F = 12.183, p < 0.001). At 15 minutes, PI reached 3.285 ± 0.262, 2.850 ± 0.285, and 2.778 ± 0.270, respectively (F = 40.617, p < 0.001). At 30 minutes, the corresponding values were 3.203 ± 0.411, 3.063 ± 0.273, and 2.815 ± 0.422 (F = 10.951, p < 0.001). Desflurane demonstrated the highest PI values at all post-induction time points, whereas sevoflurane generally showed the lowest values. HR was comparable at baseline (p = 0.086) and at 15 minutes (p = 0.440), but significant between-group differences were observed at 1, 5, 20, and 30 minutes. SBP and DBP remained comparable among the three groups throughout the observation period. MAP also showed no significant between-group differences at any assessed time point, including baseline (F = 0.693, p = 0.502), 1 minute (F = 0.133, p = 0.875), 15 minutes (F = 0.101, p = 0.904), and 30 minutes (F = 0.407, p = 0.666).

Conclusion

Desflurane, isoflurane, and sevoflurane were associated with increased PI following induction of general anaesthesia, with desflurane showing the highest PI values at most post-induction time points. These differences occurred despite broadly comparable SBP, DBP, and MAP among the three groups. PI may provide additional information about peripheral vascular tone alongside routine systemic haemodynamic monitoring; however, the observed differences in PI should not be interpreted as evidence of improved tissue perfusion or clinical superiority of one volatile anaesthetic over another.

## Introduction

Perioperative maintenance of adequate tissue perfusion is an important component of anaesthetic management because tissue hypoperfusion and resultant hypoxia may contribute to adverse outcomes in surgical patients. Surgical stress can increase oxygen demand, and inadequate tissue oxygen delivery may result in hypoxia, with severe or prolonged hypoxia potentially contributing to organ injury or death [1-3]. Anaesthetic agents can further influence cardiovascular homeostasis, making their haemodynamic and vascular effects relevant to intraoperative management [4]. Conventional systemic haemodynamic monitoring may not adequately identify changes in peripheral tissue perfusion, creating a need for feasible, continuous, and non-invasive methods of assessing peripheral perfusion [5,6].

Volatile inhalational anaesthetics produce important cardiovascular effects, including reductions in systemic vascular resistance (SVR), which may result in decreased arterial pressure [7]. Sevoflurane and isoflurane are commonly used inhalational anaesthetics with differing pharmacological effects on vascular tone. Sevoflurane has a low blood/gas partition coefficient of approximately 0.68 and is associated with rapid induction and recovery [8]. It produces dose-dependent reductions in SVR, myocardial contractility, and mean arterial pressure (MAP), with a modest increase in heart rate (HR) [9]. Isoflurane also decreases MAP through reduced SVR and has been reported to produce a greater increase in HR than sevoflurane [10]. Both agents have been associated with cerebral vasodilation [11]. Desflurane, another commonly used volatile anaesthetic, also reduces SVR and arterial pressure [7].

Differences in vasodilatory effects among volatile anaesthetics are clinically relevant because vasodilation can increase peripheral and microvascular perfusion, while excessive vasodilation may contribute to hypotension [7]. Quantitative assessment of anaesthetic-induced vasodilation has traditionally been challenging because direct assessment of vascular resistance may require invasive procedures, while calculated SVR does not exclusively represent peripheral vasomotor tone [7]. Therefore, a non-invasive indicator capable of reflecting changes in peripheral vascular tone may provide useful additional information during anaesthesia.

Perfusion index (PI), derived from the pulse oximetry signal, represents the ratio of pulsatile to non-pulsatile components of the signal and provides a non-invasive measure of peripheral perfusion [5,12]. Changes in PI reflect alterations in peripheral vascular tone, with increasing PI associated with vasodilation and increased peripheral tissue perfusion [12,13]. PI has been investigated in perioperative and critical-care settings, including assessment of anaesthetic depth, sympathetic blockade, and prediction of hypotension following spinal anaesthesia [14]. Volatile anaesthetics have also been shown to increase peripheral perfusion, and PI has been used to compare their vasodilatory effects [12].

Previous evidence indicates that volatile anaesthetics may differ in their effects on vascular tone. A randomised controlled trial comparing desflurane and sevoflurane using PI reported a more potent vasodilatory effect with desflurane, demonstrated by higher PI and lower blood pressure [7]. However, direct comparison of the vasodilatory effects of isoflurane, sevoflurane, and desflurane using a common non-invasive measure remains relevant. Therefore, the present study aims to compare the vasodilatory effects of isoflurane, sevoflurane, and desflurane using PI as the primary outcome measure, while also evaluating changes in MAP, HR, and other haemodynamic parameters to better characterise their cardiovascular effects.

## Materials and methods

Study design and setting

This prospective comparative clinical study was conducted in the Department of Anesthesiology, Shri Guru Ram Rai Institute of Medical and Health Sciences, Patel Nagar, Dehradun, India. The study was carried out over a period of 18 months, from 01 August 2024 to 31 January 2026, after obtaining approval from the Institutional Ethics Committee (IEC) (IEC Reference No. SGRR/IEC/39/23). Written informed consent was obtained from all participants before enrolment.

Sample size calculation

The sample size was estimated using data from a previously published study [15], which reported mean pre-stimulation PI values of 4.9 ± 0.8 for sevoflurane and 4.1 ± 1.0 for isoflurane. Using the formula:

$$ n = 2(Z_{\alpha/2} + Z_{\beta})^2 \frac{\sigma^2}{(\mu_1-\mu_2)^2} $$

with a two-sided α of 0.05 and 80% power, the estimated sample size was approximately 21 patients per group. The present comparative study therefore included 40 patients in each of the three groups, giving a total sample size of 120 patients(Figure 1).

**Figure 1:**
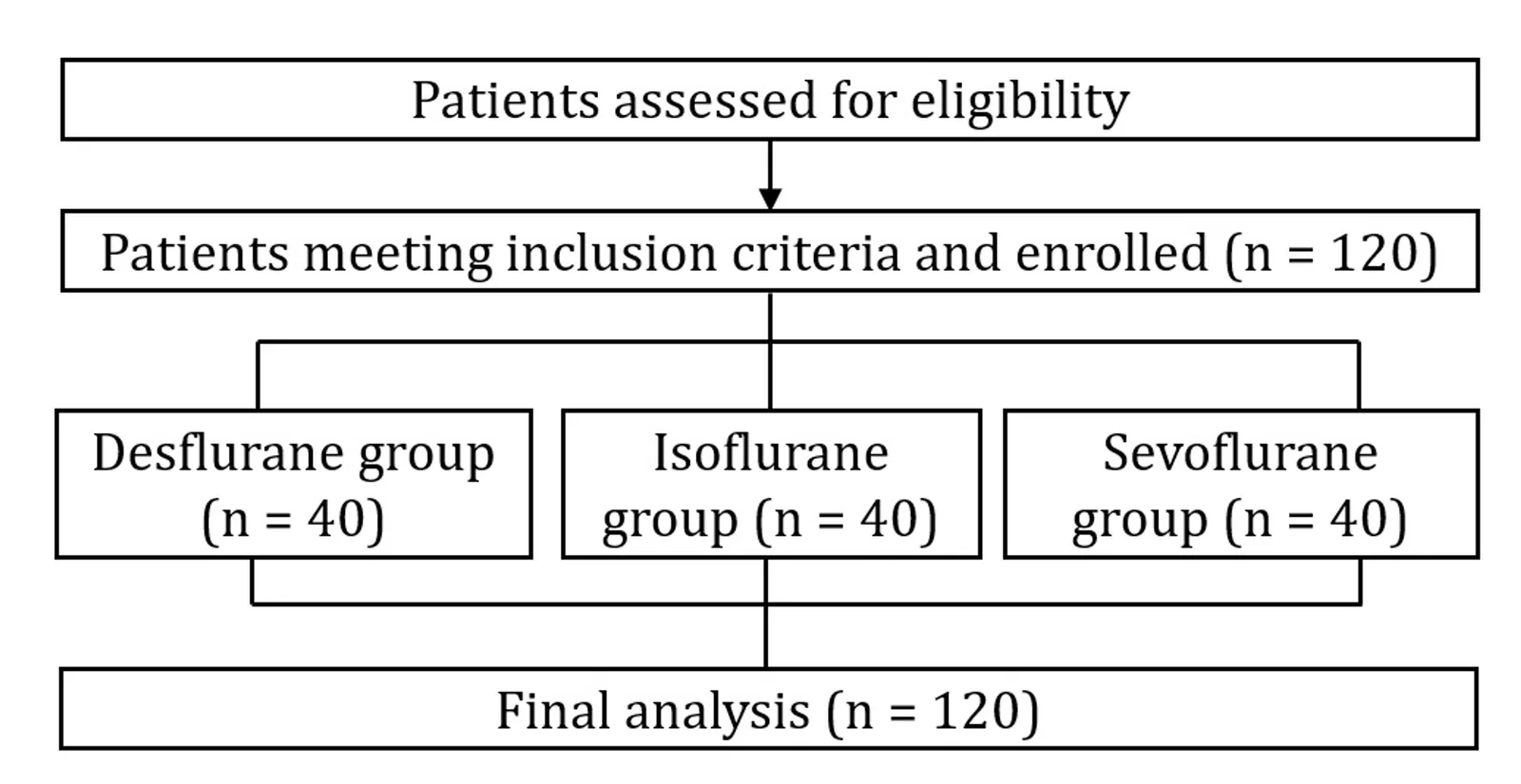
Cohort flowchart showing patient selection, eligibility assessment, enrolment, allocation to the three anaesthetic groups, and final analysis population.

Study population

The study included 120 patients of either sex, aged 18-60 years, classified as American Society of Anesthesiologists Physical Status (ASA-PS) I or II [16], and scheduled for elective surgery under general anaesthesia. Patients were categorised into three groups according to the volatile anaesthetic used for maintenance of anaesthesia: isoflurane group, sevoflurane group, and desflurane group, with 40 patients in each group.

Eligibility criteria

Patients aged 18-60 years with ASA-PS I or II who were scheduled for elective surgery under general anaesthesia were eligible for inclusion. Patients were excluded if they were unwilling to participate, were receiving medications that could affect vascular tone, including calcium channel blockers, angiotensin receptor blockers, or beta blockers, or had cardiovascular or peripheral vascular disease such as diabetic vasculopathy, Raynaud’s disease, or congestive heart failure. Patients requiring anticholinergic premedication, those with uncontrolled diabetes mellitus, and those with a history of substance abuse or chronic smoking were also excluded.

Postoperative assessment

Postoperative pain was assessed using the visual analogue scale (VAS) at 1, 2, 6, and 12 hours after surgery [17]. Time to extubation and time to discharge were recorded in minutes. Postoperative adverse events, including nausea/vomiting, dizziness, headache, and hypotension, were recorded during the postoperative observation period.

Monitoring and baseline assessment

On arrival in the operating room, standard monitoring was instituted according to ASA guidelines, including electrocardiography and blood pressure monitoring. A Dräger pulse oximeter sensor was placed on the fourth finger of the hand contralateral to the arm with the blood pressure cuff. The photoelectric plethysmographic signal obtained from the pulse oximeter was used to calculate the PI. Radial artery cannulation was performed using a 20G/22G BD arterial cannula (Becton, Dickinson and Company, Franklin Lakes, NJ) for accurate measurement of MAP. End-tidal carbon dioxide was monitored, and ventilation was adjusted to maintain an end-tidal carbon dioxide concentration of 35-45 mmHg. Core temperature was monitored using a disposable nasopharyngeal temperature sensor, and the operating room temperature was maintained at 23-24°C throughout the study. Baseline PI, MAP, and HR were recorded before induction of anaesthesia while the patient was conscious.

Anaesthetic technique

Anaesthesia was induced with intravenous fentanyl 2 µg/kg and propofol 2 mg/kg. Propofol was used only for induction and was discontinued after loss of consciousness. The volatile anaesthetic corresponding to the patient's study group, namely isoflurane, sevoflurane, or desflurane, was then administered by bag and mask. Vecuronium 1 mg/kg was administered, followed by insertion of a supraglottic airway device or endotracheal tube according to the surgical requirement. The supraglottic airway device used was LMA ProSeal™ (size 3; Teleflex Medical, Athlone, Ireland), and a Portex® plain endotracheal tube (REF GS-2002) was used when endotracheal intubation was required. Controlled ventilation was initiated with a fresh gas flow of 4 L/minute, which was maintained for 30 minutes during outcome assessment.

Maintenance of anaesthesia

The assigned volatile anaesthetic was used for maintenance of general anaesthesia. An independent anaesthesiologist adjusted the vaporiser to maintain a target end-tidal anaesthetic concentration equivalent to 1.0 minimum alveolar concentration (MAC). The vaporiser concentration was adjusted in increments or decrements of 0.1 vol% as required. No nitrous oxide or other anaesthetic adjuvant was administered during the study period. Intravenous Ringer’s lactate solution was administered at 5 mL/kg/hour.

Outcome assessment

The primary outcome was the change in peripheral PI associated with the three volatile anaesthetics. PI, HR, systolic blood pressure (SBP), diastolic blood pressure (DBP), and MAP were recorded at baseline and at 1, 5, 15, 20, and 30 minutes after induction. SpO₂ was recorded at the same time points and after extubation. The PI obtained from the pulse oximeter plethysmographic waveform was used as a non-invasive indicator of changes in peripheral vascular tone.

Statistical analysis

Data were entered into Microsoft Excel (Microsoft® Corp., Redmond, WA) and analysed using IBM SPSS Statistics for Windows, version 27.0 (IBM Corp., Armonk, NY). Quantitative variables were expressed as mean ± standard deviation (SD), while categorical variables were presented as frequency and percentage (n (%)). Differences in quantitative variables among the three groups were assessed using one-way analysis of variance (ANOVA). Categorical variables were compared using the chi-square test. A p-value < 0.05 was considered statistically significant.

## Results

The three groups were comparable at baseline. Mean age was 36.75 ± 10.37 years in the desflurane group, 36.88 ± 9.11 years in the isoflurane group, and 36.92 ± 10.42 years in the sevoflurane group (F = 0.003, p = 0.997). Mean height was 163.56 ± 5.77, 162.98 ± 4.75, and 164.41 ± 5.62 cm (F = 0.709, p = 0.494), while mean weight was 64.43 ± 6.23, 65.39 ± 6.32, and 63.45 ± 6.11 kg (F = 0.972, p = 0.381), respectively. Mean BMI was 23.43 ± 3.03, 24.15 ± 3.19, and 23.21 ± 3.05 kg/m² (F = 1.015, p = 0.365). The 18-40-year age group comprised 26 (65.0%), 28 (70.0%), and 27 (67.5%) patients, respectively, while females comprised 18 (45.0%), 19 (47.5%), and 20 (50.0%) (χ² = 0.201, p = 0.905). BMI 18.5-24.9 kg/m² was present in 29 (72.5%), 27 (67.5%), and 28 (70.0%) patients, respectively (χ² = 6.253, p = 0.181). ASA I status was observed in 24 (60.0%), 24 (60.0%), and 17 (42.5%), while ASA II status was observed in 16 (40.0%), 16 (40.0%), and 23 (57.5%), respectively (χ² = 3.290, p = 0.193). Overall, no significant between-group differences were observed for baseline characteristics (all p > 0.05), confirming good baseline comparability. Controlled ventilation was used in all patients in the three groups, with no patients receiving spontaneous ventilation (Table 1).

**Table 1:** Baseline demographic and clinical characteristics. ASA, Anesthesiologists Physical Status

Variable	Desflurane (n = 40)	Isoflurane (n = 40)	Sevoflurane (n = 40)	Test statistic	p-value
Age, years	36.75 ± 10.37	36.88 ± 9.11	36.92 ± 10.42	F = 0.003	0.997
Height, cm	163.56 ± 5.77	162.98 ± 4.75	164.41 ± 5.62	F = 0.709	0.494
Weight, kg	64.43 ± 6.23	65.39 ± 6.32	63.45 ± 6.11	F = 0.972	0.381
BMI, kg/m²	23.43 ± 3.03	24.15 ± 3.19	23.21 ± 3.05	F = 1.015	0.365
Age 18-40 years	26 (65.0%)	28 (70.0%)	27 (67.5%)	χ² = 0.228	0.892
Age 41-60 years	14 (35.0%)	12 (30.0%)	13 (32.5%)		
Female	18 (45.0%)	19 (47.5%)	20 (50.0%)	χ² = 0.201	0.905
Male	22 (55.0%)	21 (52.5%)	20 (50.0%)		
BMI 18.5-24.9	29 (72.5%)	27 (67.5%)	28 (70.0%)	χ² = 6.253	0.181
BMI 25-29.9	11 (27.5%)	10 (25.0%)	12 (30.0%)		
BMI >30	0	3 (7.5%)	0		
ASA I	24 (60.0%)	24 (60.0%)	17 (42.5%)	χ² = 3.290	0.193
ASA II	16 (40.0%)	16 (40.0%)	23 (57.5%)		
Controlled ventilation	40 (100.0%)	40 (100.0%)	40 (100.0%)	-	-
Spontaneous ventilation	0 (0%)	0 (0%)	0 (0%)	-	-

PI increased progressively after induction in all three groups. At baseline, PI was 1.055 ± 0.136, 1.118 ± 0.153, and 1.112 ± 0.179 in the desflurane, isoflurane, and sevoflurane groups, respectively, with no significant difference (F = 1.960, p = 0.145). At 1 minute, PI increased to 2.753 ± 0.377, 2.493 ± 0.241, and 2.445 ± 0.264 (F = 12.183, p < 0.001), and at 5 minutes to 3.190 ± 0.399, 2.735 ± 0.363, and 2.640 ± 0.323 (F = 26.259, p < 0.001). At 15 minutes, the values were 3.285 ± 0.262, 2.850 ± 0.285, and 2.778 ± 0.270 (F = 40.617, p < 0.001), while at 20 minutes they were 3.300 ± 0.366, 2.898 ± 0.265, and 2.848 ± 0.411 (F = 19.823, p < 0.001). At 30 minutes, PI was 3.203 ± 0.411, 3.063 ± 0.273, and 2.815 ± 0.422, respectively (F = 10.951, p < 0.001). Thus, between-group differences were significant at all post-induction time points, with the highest PI generally observed with desflurane and the lowest with sevoflurane (Table 2).

**Table 2:** Perfusion index at different time points.

Time point	Desflurane	Isoflurane	Sevoflurane	F	p-value
Baseline	1.055 ± 0.136	1.118 ± 0.153	1.112 ± 0.179	1.960	0.145
1 minute	2.753 ± 0.377	2.493 ± 0.241	2.445 ± 0.264	12.183	<0.001
5 minutes	3.190 ± 0.399	2.735 ± 0.363	2.640 ± 0.323	26.259	<0.001
15 minutes	3.285 ± 0.262	2.850 ± 0.285	2.778 ± 0.270	40.617	<0.001
20 minutes	3.300 ± 0.366	2.898 ± 0.265	2.848 ± 0.411	19.823	<0.001
30 minutes	3.203 ± 0.411	3.063 ± 0.273	2.815 ± 0.422	10.951	<0.001

Heart rate was comparable among the three groups at baseline: 77.88 ± 1.82, 78.93 ± 2.31, and 78.62 ± 2.32 bpm in the desflurane, isoflurane, and sevoflurane groups, respectively (F = 2.500, p = 0.086). At 1 minute, heart rate was 74.69 ± 1.68, 74.20 ± 2.24, and 75.79 ± 2.38 bpm (F = 5.867, p = 0.004), and at 5 minutes it was 74.38 ± 1.31, 75.56 ± 2.84, and 75.62 ± 1.95 bpm (F = 4.295, p = 0.016). At 15 minutes, values were 73.98 ± 1.18, 74.41 ± 1.90, and 74.03 ± 1.76 bpm, with no significant difference (F = 0.827, p = 0.440). Significant differences were observed at 20 minutes (72.84 ± 1.12, 74.41 ± 1.86, and 73.08 ± 1.20 bpm; F = 13.981, p < 0.001) and 30 minutes (73.14 ± 1.25, 74.18 ± 1.94, and 73.37 ± 1.40 bpm; F = 4.980, p = 0.008). Overall, HR remained comparable at baseline and 15 minutes, but differed significantly among groups at 1, 5, 20, and 30 minutes (Table 3).

**Table 3:** Heart rate at different time points.

Time point	Desflurane, mean ± SD	Isoflurane, mean ± SD	Sevoflurane, mean ± SD	F	p-value
Baseline	77.88 ± 1.82	78.93 ± 2.31	78.62 ± 2.32	2.500	0.086
1 minute	74.69 ± 1.68	74.20 ± 2.24	75.79 ± 2.38	5.867	0.004
5 minutes	74.38 ± 1.31	75.56 ± 2.84	75.62 ± 1.95	4.295	0.016
15 minutes	73.98 ± 1.18	74.41 ± 1.90	74.03 ± 1.76	0.827	0.440
20 minutes	72.84 ± 1.12	74.41 ± 1.86	73.08 ± 1.20	13.981	<0.001
30 minutes	73.14 ± 1.25	74.18 ± 1.94	73.37 ± 1.40	4.980	0.008

SBP remained comparable among the three groups throughout the study. At baseline, values were 125.16 ± 6.90, 125.07 ± 8.02, and 125.43 ± 8.48 mmHg in the desflurane, isoflurane, and sevoflurane groups, respectively (F = 0.023, p = 0.977). At 1 minute, values were 122.43 ± 5.88, 122.47 ± 6.19, and 122.21 ± 5.59 mmHg (F = 0.022, p = 0.978), and at 5 minutes, 122.06 ± 4.77, 121.19 ± 5.79, and 121.82 ± 7.18 mmHg (F = 0.227, p = 0.797). At 15 minutes, values were 121.59 ± 4.57, 121.36 ± 4.49, and 120.24 ± 5.97 mmHg (F = 0.823, p = 0.442); at 20 minutes, 118.32 ± 6.44, 118.55 ± 7.39, and 119.05 ± 5.79 mmHg (F = 0.128, p = 0.880); and at 30 minutes, 118.04 ± 4.98, 118.61 ± 6.69, and 119.18 ± 6.21 mmHg (F = 0.364, p = 0.696). Post-extubation values were 124.70 ± 3.06, 124.61 ± 3.93, and 124.78 ± 4.06 mmHg (F = 0.019, p = 0.981). Thus, no significant between-group differences were observed at any time point (all p > 0.05) (Table 4).

**Table 4:** Systolic blood pressure (mmHg) at different time points.

Time point	Desflurane, mean ± SD	Isoflurane, mean ± SD	Sevoflurane, mean ± SD	F	p-value
Baseline	125.16 ± 6.90	125.07 ± 8.02	125.43 ± 8.48	0.023	0.977
1 minute	122.43 ± 5.88	122.47 ± 6.19	122.21 ± 5.59	0.022	0.978
5 minutes	122.06 ± 4.77	121.19 ± 5.79	121.82 ± 7.18	0.227	0.797
15 minutes	121.59 ± 4.57	121.36 ± 4.49	120.24 ± 5.97	0.823	0.442
20 minutes	118.32 ± 6.44	118.55 ± 7.39	119.05 ± 5.79	0.128	0.880
30 minutes	118.04 ± 4.98	118.61 ± 6.69	119.18 ± 6.21	0.364	0.696
Post-extubation	124.70 ± 3.06	124.61 ± 3.93	124.78 ± 4.06	0.019	0.981

DBP remained comparable among the three groups at all time points. At baseline, values were 78.30 ± 5.07, 78.90 ± 6.55, and 77.78 ± 5.10 mmHg in the desflurane, isoflurane, and sevoflurane groups, respectively (F = 0.398, p = 0.672). At 1 minute, values were 75.03 ± 3.91, 75.59 ± 5.34, and 75.23 ± 4.06 mmHg (F = 0.162, p = 0.851), and at 5 minutes, 75.20 ± 3.26, 76.12 ± 5.11, and 75.44 ± 3.90 mmHg (F = 0.525, p = 0.593). At 15 minutes, values were 76.99 ± 3.13, 74.83 ± 4.79, and 75.56 ± 4.43 mmHg (F = 2.775, p = 0.066). At 20 minutes, values were 74.95 ± 3.25, 74.84 ± 5.07, and 74.18 ± 4.05 mmHg (F = 0.398, p = 0.673), while at 30 minutes, they were 74.46 ± 4.31, 73.96 ± 4.72, and 74.35 ± 3.32 mmHg (F = 0.163, p = 0.850). Post-extubation values were 82.23 ± 2.52, 82.45 ± 3.18, and 82.82 ± 2.69 mmHg (F = 0.447, p = 0.641). Overall, no significant between-group differences were observed at any time point (all p > 0.05) (Table 5).

**Table 5:** Diastolic blood pressure (mmHg) at different time points.

Time point	Desflurane, mean ± SD	Isoflurane, mean ± SD	Sevoflurane, mean ± SD	F	p-value
Baseline	78.30 ± 5.07	78.90 ± 6.55	77.78 ± 5.10	0.398	0.672
1 minute	75.03 ± 3.91	75.59 ± 5.34	75.23 ± 4.06	0.162	0.851
5 minutes	75.20 ± 3.26	76.12 ± 5.11	75.44 ± 3.90	0.525	0.593
15 minutes	76.99 ± 3.13	74.83 ± 4.79	75.56 ± 4.43	2.775	0.066
20 minutes	74.95 ± 3.25	74.84 ± 5.07	74.18 ± 4.05	0.398	0.673
30 minutes	74.46 ± 4.31	73.96 ± 4.72	74.35 ± 3.32	0.163	0.850
Post-extubation	82.23 ± 2.52	82.45 ± 3.18	82.82 ± 2.69	0.447	0.641

MAP remained comparable across the three groups at all time points. At baseline, MAP was 94.34 ± 5.29, 93.07 ± 4.41, and 93.46 ± 5.06 mmHg in the desflurane, isoflurane, and sevoflurane groups, respectively (F = 0.693, p = 0.502). At 1 minute, values were 91.76 ± 4.48, 91.38 ± 4.31, and 91.86 ± 4.37 mmHg (F = 0.133, p = 0.875), and at 5 minutes, 92.96 ± 3.67, 91.74 ± 4.26, and 90.65 ± 4.55 mmHg (F = 3.045, p = 0.051). At 15 minutes, MAP was 91.14 ± 3.92, 91.55 ± 4.44, and 91.35 ± 3.88 mmHg (F = 0.101, p = 0.904); at 20 minutes, 89.43 ± 4.31, 90.61 ± 3.94, and 90.43 ± 3.64 mmHg (F = 1.038, p = 0.358); and at 30 minutes, 90.71 ± 3.92, 89.88 ± 4.10, and 90.13 ± 4.54 mmHg (F = 0.407, p = 0.666). Post-extubation values were 96.04 ± 3.18, 95.73 ± 3.24, and 95.40 ± 3.31 mmHg (F = 0.380, p = 0.685). Overall, no significant between-group differences were observed at any time point, although the 5-minute difference approached significance (p = 0.051) (Table 6).

**Table 6:** Mean arterial pressure (mmHg) at different time points.

Time point	Desflurane, mean ± SD	Isoflurane, mean ± SD	Sevoflurane, mean ± SD	F	p-value
Baseline	94.34 ± 5.29	93.07 ± 4.41	93.46 ± 5.06	0.693	0.502
1 minute	91.76 ± 4.48	91.38 ± 4.31	91.86 ± 4.37	0.133	0.875
5 minutes	92.96 ± 3.67	91.74 ± 4.26	90.65 ± 4.55	3.045	0.051
15 minutes	91.14 ± 3.92	91.55 ± 4.44	91.35 ± 3.88	0.101	0.904
20 minutes	89.43 ± 4.31	90.61 ± 3.94	90.43 ± 3.64	1.038	0.358
30 minutes	90.71 ± 3.92	89.88 ± 4.10	90.13 ± 4.54	0.407	0.666
Post-extubation	96.04 ± 3.18	95.73 ± 3.24	95.40 ± 3.31	0.380	0.685

Oxygen saturation was comparable across groups at baseline, with SpO₂ values of 98.107 ± 0.225%, 98.053 ± 0.277%, and 98.188 ± 0.415% in the desflurane, isoflurane, and sevoflurane groups, respectively (F = 1.845, p = 0.163). At 1 minute, values were 97.970 ± 0.107%, 97.988 ± 0.196%, and 98.085 ± 0.074% (F = 8.324, p < 0.001). No significant differences were observed at 5 minutes (97.940 ± 0.081%, 97.928 ± 0.208%, and 97.945 ± 0.082%; F = 0.173, p = 0.841), 15 minutes (97.848 ± 0.124%, 97.902 ± 0.236%, and 97.890 ± 0.115%; F = 1.184, p = 0.310), or 20 minutes (97.715 ± 0.077%, 97.768 ± 0.213%, and 97.700 ± 0.072%; F = 2.674, p = 0.073). Significant differences were observed at 30 minutes, with values of 97.557 ± 0.068%, 97.818 ± 0.252%, and 97.525 ± 0.150% (F = 34.070, p < 0.001), and post-extubation, with values of 97.640 ± 0.078%, 97.505 ± 0.279%, and 97.380 ± 0.247% (F = 13.983, p < 0.001). Thus, SpO₂ was comparable at baseline, 5, 15, and 20 minutes, but significant between-group differences were observed at 1 and 30 minutes and post-extubation (Table 7).

**Table 7:** Oxygen saturation (SpO₂) at different time points.

Time point	Desflurane	Isoflurane	Sevoflurane	F	p-value
Baseline	98.107 ± 0.225	98.053 ± 0.277	98.188 ± 0.415	1.845	0.163
1 minute	97.970 ± 0.107	97.988 ± 0.196	98.085 ± 0.074	8.324	<0.001
5 minutes	97.940 ± 0.081	97.928 ± 0.208	97.945 ± 0.082	0.173	0.841
15 minutes	97.848 ± 0.124	97.902 ± 0.236	97.890 ± 0.115	1.184	0.310
20 minutes	97.715 ± 0.077	97.768 ± 0.213	97.700 ± 0.072	2.674	0.073
30 minutes	97.557 ± 0.068	97.818 ± 0.252	97.525 ± 0.150	34.070	<0.001
Post-extubation	97.640 ± 0.078	97.505 ± 0.279	97.380 ± 0.247	13.983	<0.001

Postoperative VAS scores were significantly lower in the Desflurane group at all time points. At 1 hour, mean VAS was 3.175 ± 0.119, 3.492 ± 0.114, and 3.600 ± 0.134 in the desflurane, isoflurane, and sevoflurane groups, respectively (F = 129.644, p < 0.001). At 2 hours, scores were 2.898 ± 0.139, 3.112 ± 0.124, and 3.152 ± 0.124 (F = 45.065, p < 0.001); at 6 hours, 2.308 ± 0.094, 2.530 ± 0.176, and 2.590 ± 0.130 (F = 46.958, p < 0.001); and at 12 hours, 1.830 ± 0.082, 1.990 ± 0.063, and 1.998 ± 0.131, respectively (F = 38.445, p < 0.001). Mean time to extubation was similar across groups (10.06 ± 1.08, 10.21 ± 1.05, and 10.30 ± 0.71 minutes; F = 0.628, p = 0.535). However, time to discharge differed significantly, at 49.50 ± 2.19, 51.44 ± 2.45, and 50.78 ± 1.56 minutes, respectively (F = 8.814, p < 0.001). Overall, desflurane was associated with lower postoperative VAS scores, while time to extubation was comparable and time to discharge differed significantly among the groups (Table 8).

**Table 8:** Postoperative pain and recovery outcomes.

Outcome/time point	Desflurane	Isoflurane	Sevoflurane	F	p-value
VAS 1 hour	3.175 ± 0.119	3.492 ± 0.114	3.600 ± 0.134	129.644	<0.001
VAS 2 hours	2.898 ± 0.139	3.112 ± 0.124	3.152 ± 0.124	45.065	<0.001
VAS 6 hours	2.308 ± 0.094	2.530 ± 0.176	2.590 ± 0.130	46.958	<0.001
VAS 12 hours	1.830 ± 0.082	1.990 ± 0.063	1.998 ± 0.131	38.445	<0.001
Time to extubation	10.06 ± 1.08	10.21 ± 1.05	10.30 ± 0.71	0.628	0.535
Time to discharge	49.50 ± 2.19	51.44 ± 2.45	50.78 ± 1.56	8.814	<0.001

Postoperative adverse events were infrequent and did not differ significantly among the three groups. Nausea/vomiting occurred in two (5.0%) patients in the desflurane group, three (7.5%) in the isoflurane group, and four (10.0%) in the sevoflurane group (χ² = 0.721, p = 0.697). Dizziness was reported in three (7.5%), two (5.0%), and two (5.0%) patients, respectively (χ² = 0.303, p = 0.859). Headache occurred in one (2.5%), one (2.5%), and two (5.0%) patients in the desflurane, isoflurane, and sevoflurane groups, respectively (χ² = 0.517, p = 0.772). Hypotension was reported in one (2.5%), two (5.0%), and three (7.5%) patients, respectively (χ² = 1.053, p = 0.591). Overall, no statistically significant differences were observed in the incidence of nausea/vomiting, dizziness, headache, or hypotension among the three groups (all p > 0.05) (Table 9).

**Table 9:** Postoperative adverse events.

Adverse event	Desflurane, n (%)	Isoflurane, n (%)	Sevoflurane, n (%)	Pearson χ²	p-value
Nausea/vomiting	2 (5.0)	3 (7.5)	4 (10.0)	0.721	0.697
Dizziness	3 (7.5)	2 (5.0)	2 (5.0)	0.303	0.859
Headache	1 (2.5)	1 (2.5)	2 (5.0)	0.517	0.772
Hypotension	1 (2.5)	2 (5.0)	3 (7.5)	1.053	0.591

## Discussion

In the present study, the three groups were comparable at baseline, with no significant differences in age, height, weight, BMI, sex distribution, BMI category, or ASA status (all p > 0.05). Mean age was 36.75 ± 10.37, 36.88 ± 9.11, and 36.92 ± 10.42 years, and mean BMI was 23.43 ± 3.03, 24.15 ± 3.19, and 23.21 ± 3.05 kg/m² in the desflurane, isoflurane, and sevoflurane groups, respectively. This comparable baseline profile is consistent with previous studies. Ibnul et al. reported similar age, height, weight, BMI, and ASA distribution between isoflurane and sevoflurane groups [18]. Oh et al. also found well-balanced baseline characteristics between desflurane and sevoflurane after propensity score matching [19]. Kowalczyk et al. reported comparable age and body weight between the propofol and desflurane groups [20]. Similarly, Ryu et al. found comparable demographic and baseline characteristics between desflurane and sevoflurane groups [7]. Jones et al. also reported similar baseline characteristics between sevoflurane and isoflurane groups [21]. Ajayan et al. found no significant differences in age, height, weight, BMI, or ASA status between isoflurane and sevoflurane groups [15]. Overall, these findings support the comparability of the study groups at baseline and reduce the likelihood that differences in subsequent outcomes were related to baseline demographic characteristics.

In the present study, PI increased after induction in all three groups. Baseline PI was 1.055 ± 0.136, 1.118 ± 0.153, and 1.112 ± 0.179 in the desflurane, isoflurane, and sevoflurane groups, respectively (F = 1.960, p = 0.145). PI was significantly higher with desflurane at 1, 5, 15, 20, and 30 minutes (all p < 0.001), reaching 2.753 ± 0.377, 3.190 ± 0.399, 3.285 ± 0.262, 3.300 ± 0.366, and 3.203 ± 0.411, respectively. These findings are consistent with Ibnul et al., who reported higher PI with isoflurane than sevoflurane from 15 minutes onward [18]. Oh et al. similarly found higher intraoperative PI with desflurane than sevoflurane (3.1 (2.0-4.2) vs. 2.6 (1.8-3.8), p = 0.002), supporting the higher PI observed with desflurane in the present study [19]. Ryu et al. also reported significantly higher PI with desflurane than sevoflurane before and after nociceptive stimulation (p < 0.001), further supporting our findings [7]. Kowalczyk et al. observed a significant increase in PI during surgery with desflurane, although the difference between desflurane and propofol was not significant (p = 0.240), which is partly consistent with the present finding of increased PI after induction [20]. In contrast, Ajayan et al. found no significant difference in PI between isoflurane and sevoflurane before or after nociceptive stimulation (p = 0.891 and p = 0.167, respectively), which differs from the significant difference observed between these agents in the present study [15].

In the present study, HR remained broadly comparable among the three groups, with significant differences observed at 1, 5, 20, and 30 minutes. Baseline HR was 77.88 ± 1.82, 78.93 ± 2.31, and 78.62 ± 2.32 bpm in the desflurane, isoflurane, and sevoflurane groups, respectively (F = 2.500, p = 0.086), while at 15 minutes it was 73.98 ± 1.18, 74.41 ± 1.90, and 74.03 ± 1.76 bpm, respectively (F = 0.827, p = 0.440). These findings are consistent with Ibnul et al., who reported comparable HRs between isoflurane and sevoflurane at baseline and most time points, although HR was higher with isoflurane after nociception (90.40 ± 5.07 vs. 87.33 ± 4.59 bpm, p = 0.017) [18]. Oh et al. also found no significant difference between desflurane and sevoflurane (71.1 (64.3-79.4) vs. 69.4 (61.9-77.2) bpm, p = 0.168), supporting our findings [19]. Kowalczyk et al. reported higher HR with desflurane at selected time points (p < 0.001), partly supporting the significant differences observed in our study [20]. Ryu et al. found similar HR between desflurane and sevoflurane before and after stimulation, further supporting our findings [7]. Ajayan et al. also reported comparable pre-stimulus HR, although HR was higher with isoflurane after nociception (103.11 ± 15.17 vs. 95.42 ± 9.08 beats/minute, p = 0.047) [15]. Overall, previous studies largely support the comparable HR response observed in the present study, with differences mainly appearing during specific time points or after nociceptive stimulation.

In our study, SBP remained comparable among the three groups throughout the study period. Baseline SBP was 125.16 ± 6.90, 125.07 ± 8.02, and 125.43 ± 8.48 mmHg in the desflurane, isoflurane, and sevoflurane groups, respectively (F = 0.023, p = 0.977). No significant differences were observed at any intraoperative time point or after extubation (p > 0.05). These findings are consistent with Ibnul et al., who reported comparable SBP between isoflurane and sevoflurane at baseline and throughout the observation period, including before and after nociceptive stimulation (all p > 0.05) [18]. Overall, these findings support the stability of SBP across different volatile anaesthetic agents.

In the current study, DBP remained comparable among the three groups throughout the study period. Baseline DBP was 78.30 ± 5.07, 78.90 ± 6.55, and 77.78 ± 5.10 mmHg in the desflurane, isoflurane, and sevoflurane groups, respectively (F = 0.398, p = 0.672). No significant differences were observed at any intraoperative time point or after extubation (p > 0.05). These findings are consistent with Ibnul et al., who reported comparable DBP between isoflurane and sevoflurane at baseline and most time points (p > 0.05). However, they observed a significant increase in DBP with isoflurane after nociception compared with sevoflurane (83.00 ± 5.29 vs. 78.33 ± 7.58 mmHg, p = 0.019), which was not observed in the present study [18]. Overall, the findings support a largely comparable DBP response between volatile anaesthetic agents.

In this study, MAP remained comparable among the three groups throughout the study period. Baseline MAP was 94.34 ± 5.29, 93.07 ± 4.41, and 93.46 ± 5.06 mmHg in the desflurane, isoflurane, and sevoflurane groups, respectively (F = 0.693, p = 0.502). No significant differences were observed at any intraoperative time point or after extubation (p > 0.05). These findings are consistent with Oh et al., who reported similar MAP between desflurane and sevoflurane (80.2 (74.9-88.5) vs. 80.5 (74.6-86.9) mmHg, p = 0.681) [19]. Kowalczyk et al. also found only small differences in arterial pressure at selected time points, with no clinically relevant effect [20]. Ajayan et al. similarly reported comparable MAP between isoflurane and sevoflurane before and after nociceptive stimulation (p = 0.172 and p = 0.390, respectively) [15]. In contrast, Ryu et al. reported significantly lower MAP with desflurane than sevoflurane both before and after nociceptive stimulation (p < 0.001), which differs from the present findings [7]. Overall, most available studies support the comparable MAP response observed in the present study.

In our study, postoperative pain scores were significantly lower in the desflurane group at 1, 2, 6, and 12 hours (all p < 0.001), with mean VAS scores of 3.175 ± 0.119, 2.898 ± 0.139, 2.308 ± 0.094, and 1.830 ± 0.082, respectively. Time to extubation was comparable among the three groups (10.06 ± 1.08, 10.21 ± 1.05, and 10.30 ± 0.71 minutes; F = 0.628, p = 0.535), while time to discharge was significantly shorter in the Desflurane group (49.50 ± 2.19, 51.44 ± 2.45, and 50.78 ± 1.56 minutes; F = 8.814, p < 0.001). Jones et al. similarly found comparable extubation times between sevoflurane and isoflurane (5.2 (3.6-9.3) vs. 5.3 (3.7-8.3) hours, p = 0.95), as well as similar ICU and hospital discharge times (p = 0.17 and p = 0.86, respectively), supporting the comparable recovery observed between anaesthetic groups in the present study [21].

In this study, postoperative adverse events were infrequent and comparable among the three groups. Nausea/vomiting occurred in two (5.0%), three (7.5%), and four (10.0%) patients (χ² = 0.721, p = 0.697), dizziness in three (7.5%), two (5.0%), and two (5.0%) patients (χ² = 0.303, p = 0.859), headache in one (2.5%), one (2.5%), and two (5.0%) patients (χ² = 0.517, p = 0.772), and hypotension in one (2.5%), two (5.0%), and three (7.5%) patients in the desflurane, isoflurane, and sevoflurane groups, respectively (χ² = 1.053, p = 0.591). Jones et al. similarly reported no significant differences in major postoperative outcomes between sevoflurane and isoflurane, including prolonged ICU stay, mortality, vasopressor use, haemodialysis, atrial fibrillation, stroke, and ICU readmission (p > 0.05), supporting the comparable postoperative safety profile observed in our study [21].

This study has certain limitations. It was conducted at a single centre and included a relatively small sample of 120 patients, which may limit the generalizability of the findings to broader surgical populations. The study included only patients aged 18-60 years with ASA-PS I or II, limiting applicability to older patients and those with greater comorbidity. PI was assessed over a relatively short intraoperative period of 30 minutes; therefore, longer term effects of the three anaesthetic agents on peripheral perfusion were not evaluated. In addition, the study was limited to elective surgical patients, and the findings may not be applicable to emergency or high-risk surgical settings. The type and intensity of surgical stimulation were not standardised and may have influenced sympathetic activity and cardiovascular parameters, including PI. Future studies involving more homogeneous surgical populations or standardised experimental conditions may help better isolate the effects of individual volatile anaesthetic agents on PI.

The clinical relevance of the observed differences in PI should also be interpreted with caution. PI is a non-invasive indicator of peripheral vascular tone and may provide additional information alongside routine systemic haemodynamic monitoring. In the present study, the higher PI observed with desflurane was accompanied by broadly comparable SBP, DBP, and MAP among the three groups. However, this study was not designed to determine whether differences in PI translate into improved tissue perfusion or clinically meaningful patient outcomes. Therefore, PI should be considered an adjunctive monitoring parameter rather than a standalone measure for selecting a particular volatile anaesthetic. Although desflurane showed the highest PI values in this study, these findings alone do not provide sufficient evidence to recommend desflurane over isoflurane or sevoflurane. Further studies assessing the relationship between PI, tissue perfusion, and clinical outcomes are warranted.

## Conclusions

The present study demonstrated that desflurane, isoflurane, and sevoflurane were associated with increased PI following induction of general anaesthesia, with desflurane showing the highest PI values at most post-induction time points. However, these differences in PI were not accompanied by significant differences in SBP, DBP, or MAP. PI may therefore provide additional information about changes in peripheral vascular tone that may not be apparent from routine systemic haemodynamic measurements. However, because PI is an indirect measure of peripheral perfusion and the present study did not assess clinical outcomes related to tissue perfusion, the observed differences should not be interpreted as evidence that one volatile anaesthetic is clinically superior to another. The choice of volatile anaesthetic should therefore continue to be based on the overall clinical context and patient characteristics.
